# Association between Polymorphisms in Lysyl Oxidase-Like 1 and Susceptibility to Pseudoexfoliation Syndrome and Pseudoexfoliation Glaucoma

**DOI:** 10.1371/journal.pone.0090331

**Published:** 2014-03-06

**Authors:** Jun-Zhou Tang, Xiu-Qing Wang, Fa-Feng Ma, Bo Wang, Peng-Fei Wang, Zhi-Xi Peng, Xi-Yuan Zhou

**Affiliations:** 1 Center of Bone Metabolism and Repair, State Key Laboratory of Trauma, Burns and Combined injury, Trauma Center, Research Institute of Surgery, Daping Hospital, Third Military Medical University, Chongqing, China; 2 Department of Ophthalmology, Second Affiliated Hospital, Chongqing University of Medical Sciences, Chongqing, China; 3 Department of Endocrinology, Second Affiliated Hospital, Chongqing Medical University, Chongqing, P.R. China; 4 Department of Emergency, Second Affiliated Hospital, Chongqing Medical University, Chongqing, P.R. China; Tor Vergata University of Rome, Italy

## Abstract

The present knowledge on the association of single nucleotide polymorphisms (SNPs) of lysyl oxidase-like 1 (LOXL1) with pseudoexfoliation syndrome (PEXS) and pseudoexfoliation glaucoma (PEXG) is controversial and inconclusive. This meta-analysis sought to derive a more precise estimation of the effects of LOXL1 SNP loci (rs1048661, rs3825942, and rs2165241) on PEXS/PEXG. Literature searches were conducted on the PubMed, EMBASE, ISI Web of Science, and Cochrane Library databases through October 2013. Twelve studies describing 1810 cases and 1790 controls met the inclusion criteria. The strengths of the associations found through the meta-analysis were assessed with pooled odds ratios and their 95% confidence intervals (CI). A meta-regression analysis was also used to examine the influence of the study and population characteristics. The results indicated that rs1048661 TT carriers had 92.1% and 40.4% less risk of developing PEXS/PEXG than did the controls in the Caucasian and Asian populations, respectively. Carriers of rs3825942 AA or rs2165241 CC also had significantly less PEXS/PEXG susceptibility than did the non-carriers. Meta-regression showed that in Caucasians, the male proportion (slope: 0.272; 95% CI: 0.167–0.376; P = 0.0001) and mean age (slope: 0.796; 95% CI: 0.375–1.217; P = 0.0002) of the PEXS/PEXG subjects correlated positively with the effect of rs3825942 on PEXS/PEXG susceptibility. The meta-analysis suggested that LOXL1 rs1048661 TT, rs3825942 AA, and rs2165241 CC were associated with a reduced risk of developing PEXS/PEXG.

## Introduction

Pseudoexfoliation syndrome (PEXS) is an age-related systemic disorder that is the most common cause of secondary glaucoma worldwide and the most frequent cause of unilateral glaucoma [1], [2]. In addition, PEXS progresses to pseudoexfoliation glaucoma (PEXG), which responds poorly to medical therapy in comparison to other types of glaucoma and can lead to the rapid progression of optic nerve damage [3].

Despite its worldwide distribution, there is a clear tendency for PEXS to cluster geographically and in certain racial or ethnic subgroups [4]. Furthermore, PEXS has a strong familial association [5]. The underlying causes of the different prevalence rates between age-matched geographical and ethnic populations remain unknown, but appear to be related to variation in genetic background [5], [6]. Lysyl oxidase-like 1 (LOXL1) is a member of the lysyl oxidase gene family and is essential to the biogenesis of connective tissue [7]; it encodes an extracellular copper-dependent amine oxidase that catalyzes the first step in the formation of crosslinks in collagens and elastin [7], [8]. A highly conserved amino acid sequence at the C-terminus appears to be sufficient for amine oxidase activity, suggesting that all family members may retain this function. A fibrillar, proteinaceous substance, is produced in abnormally high concentrations within the ocular tissues of patients with PEXG, and LOXL1 may be relevant to its formation [9], [10].

A genome-wide association study identified three common single nucleotide polymorphisms (SNPs) in the LOXL1 gene on chromosome 15q24.1 that were strongly associated with pseudoexfoliation syndrome [11]. The LOXL1 polymorphisms included one intronic SNP, rs2165241, located within the first intron, and two non-synonymous coding SNPs, rs1048661 and rs3825942, located within the first exon. The association of LOXL1 with PEXS/PEXG has recently been confirmed in several different populations [12], [13], [14], [15], [16]. However, the reported associations are controversial and inconclusive due to factors including the limited sample sizes,different ethnicities, and genotyping procedures [17], [18]. Believing a meta-analysis would provide more credible evidence by systematically summarizing the existing data, we gathered eligible studies to investigate the association between the LOXL1 gene polymorphisms and susceptibility to PEXS/PEXG.

## Materials and Methods

### Search strategy

The PubMed, EMBASE, ISI Web of Science, and the Cochrane Library databases were electronically searched for case-control studies published through October 2013 that examined the association of the LOXL1 gene polymorphisms with the PEXS/PEXG susceptibility. The search strategy was based on a combination of “(lysyl oxidase-like 1 OR LOXL1) AND (gene OR variants OR polymorphism OR alleles OR mutation) AND (pseudoexfoliation syndrome OR pseudoexfoliation glaucoma)”. We also manually searched references in key articles. The language was limited to English.

### Selection criteria

The inclusion criteria were as follows: (a) evaluation of the association of the LOXL1 gene polymorphisms with the PEXS/PEXG risk; (b) case-control studies; (c) sufficient published data for estimating an odds ratio (OR) with a 95% confidence interval (CI); and (d) PEXS was diagnosed as the presence of pseudoexfoliative material on the anterior lens capsule after maximal pupil dilatation. PEXG was diagnosed if patients had typical features of PXFS and all of the following: an initial intraocular pressure of at least 22 mm Hg, glaucomatous optic disc changes, visual field defects consistentent with optic nerve damage, and no evidence of other conditions causing secondary glaucoma [19].The study authors were contacted for supplemental data if the information was not available in the publication. The studies with overlapping patient samples were excluded, and only the studies with the larger numbers of patients were included. Two of the authors independently identified and reviewed each relevant paper, and disagreements were reconciled through group discussion.

### Data extraction

To show the relationship between the LOXL1 gene polymorphisms and PEXS/PEXG risk, the most strongly and independently associated SNPs were selected for the analysis (rs1048661, rs3825942, and rs2165241). Information regarding the following aspects was retrieved from each study according to a fixed protocol: study design; geographical location; population ethnicity, definition and numbers of cases and controls; DNA extraction and genotyping methods; frequency of the genotypes; mean age of the patients; and proportion of the patients who were male. When the studies included subjects of more than one ethnicity, the genotype data were extracted separately for each ethnic group. The genetic equilibrium of the LOXL1 gene for the control group of each study was evaluated by testing for Hardy-Weinberg equilibrium (HWE) using chi-square analyses [20]. A state of disequilibrium was defined as a P value <0.05.

### Statistical analysis

A summary OR with a 95% CI was calculated to assess the strength of the association of the LOXL1 gene polymorphisms with the PEXS/PEXG risk. The OR of each study was first calculated in a 2×2 table. Pooled ORs for the risk were then calculated for the allele frequency comparison and the additive, dominant, and recessive models. The between-study heterogeneity was evaluated with a Q statistic, and a P value <0.1 was considered statistically significant [21]. If the P value was >0.1, a fixed-effect model was used for the meta-analysis; otherwise, a random-effect model was used. The significance of the pooled OR was determined with the Z-test, and P<0.05 was considered statistically significant.

To consider the possible sources of heterogeneity, we stratified the studies by ethnicity and repeated the analysis separately for each group. Furthermore, we performed a meta-regression analysis to assess the influence of the population characteristics [22]. We also performed sensitivity analyses, serially excluding studies to determine the sources of heterogeneity and assess the stability of the results. The Begg funnel plot asymmetry wasassessed with Egger linear regression tests, a linear regression approach that measures funnel plot asymmetry on the natural logarithm scale of the OR. The significance of the intercept was determined by the *t*-test suggested by Egger, with P<0.05 considered to represent statistically significant publication bias [23].

All calculations were performed using the Comprehensive Meta-Analysis computer program version 2 (Biostat, Englewood, NJ, USA).

## Results

### Characteristics of the articles in the analysis

**Figure 1:**
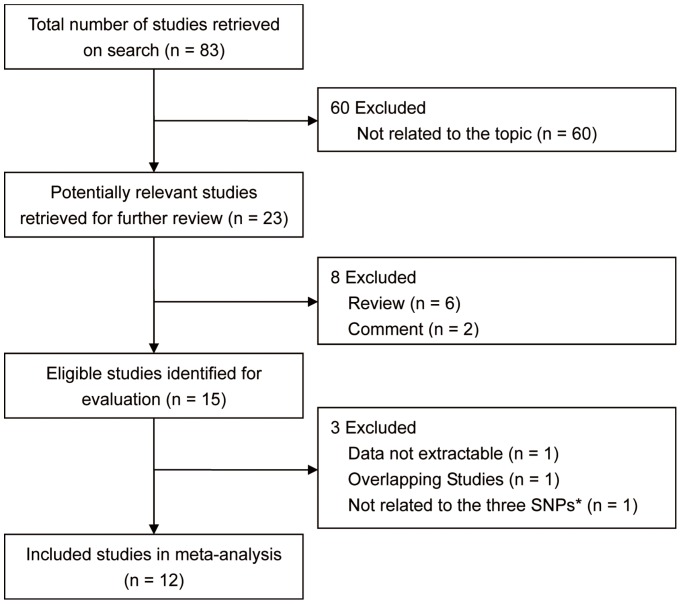
Flowchart of the study selection. SNPs, single nucleotide polymorphisms.

The first search retrieved 83 articles. After eliminating the 60 studies not related to the topic, 23 potentially relevant studies were identified for further evaluation. In these studies, the titles or abstracts indicated that they evaluated the association between the LOXL1 gene polymorphisms and PEXS/PEXG susceptibility; however, some studies did not meet all of the study inclusion criteria. Ultimately, 11 studies were excluded because they were undesirable article types (review or letter; n = 8), had overlapping patient samples (n = 1) [24], insufficient data (n = 1) [25], or were not related to the three SNPs (n = 1) [26]. Finally, 12 studies with a total of 1810 cases and 1790 controls were included in the analysis. Figure 1 shows the flowchart for the study selection.

Table 1 shows the primary characteristics of the studies included in this meta-analysis. All of the studies used healthy control subjects, and the sample sizes ranged from 66 to 1133. The studies originated from Europe (n = 5) [12], [14], [27], [28], [29], Asia (n = 6) [13], [15], [17], [18], [30], [31], and Latin America (n = 1) [16]. Two studies extracted only the allele frequencies [12], [17]. The study by Park et al. [18] represented the data as (minor homozygosity + heterozygosity)/(major homozygosity). There were no significant differences between the case and control subjects with respect to age distribution.

**Table 1:** Main characteristics of all studies included in the meta-analysis.

First author (year)	Population ethnicity	LOXL1 dpSNP rsID, Allele	PEXS/PEXG	Controls	Case males n (%)	Case mean age (year)	HWE*
Challa (2008)	Caucasian	rs1048661 T/G					YES
		rs3825942 A/G	50	235	39 (78.0)	74.0	YES
		rs2165241 C/T					YES
Fan (2008)	Caucasian	rs1048661 T/G					NO
		rs3825942 A/G	199	116	84 (42.2)	75.0	NO
		rs2165241 C/T					YES
Ozaki (2008)	Asian	rs1048661 T/G					YES
		rs3825942 A/G	209	172	67 (32.1)	78.0	YES
		rs2165241 C/T					YES
Pasutto (2008)	Caucasian	rs1048661 T/G					YES
		rs3825942 A/G	726	412	312 (43.0)	77.1	NO
		rs2165241 C/T					NO
Ramprasad (2008)	Asian	rs1048661 T/G	52	97	27 (51.9)	68.9	YES
		rs3825942 A/G					YES
Lee (2009)	Asian	rs1048661 T/G	62	171	30 (48.4)	74.7	YES
		rs3825942 A/G					YES
Abu-Amero (2010)	Asian	rs1048661 T/G	93	101	61 (65.6)	72.3	YES
		rs3825942 A/G					YES
Malukiewicz (2011)	Caucasian	rs1048661 T/G					YES
		rs3825942 A/G	36	30	9 (25.0)	73.0	YES
		rs2165241 C/T					YES
Jaimes (2012)	Latin American	rs1048661 T/G					NO
		rs3825942 A/G	102	97	NA	74.8	YES
		rs2165241 C/T					YES
Micheal (2012)	Asian	rs1048661 T/G	128	180	69 (53.9)	47.3	YES
		rs3825942 A/G					YES
Metaxaki (2013)	Caucasian	rs1048661 T/G					YES
		rs3825942 A/G	48	52	43 (49.4)	77.5	NO
		rs2165241 C/T					YES
Park (2013)	Asian	rs1048661 T/G					YES
		rs3825942 A/G	110	127	53 (47.3)	71.6	YES
		rs2165241 C/T					YES

HWE, Hardy-Weinberg equilibrium; LOXL1, lysyl oxidase-like 1; NA, not available; PEXG, pseudoexfoliation glaucoma; PEXS, pseudoexfoliation syndrome; SNP, single nucleotide polymorphism.

* The genetic equilibrium of the LOXL1 gene for the control group of each study was evaluated by testing for HWE using chi-square analyses. Disequilibrium was defined as P<0.05.

### Quantitative synthesis

#### Association of the rs1048661 T/G polymorphism with PEXS/PEXG

**Figure 2:**
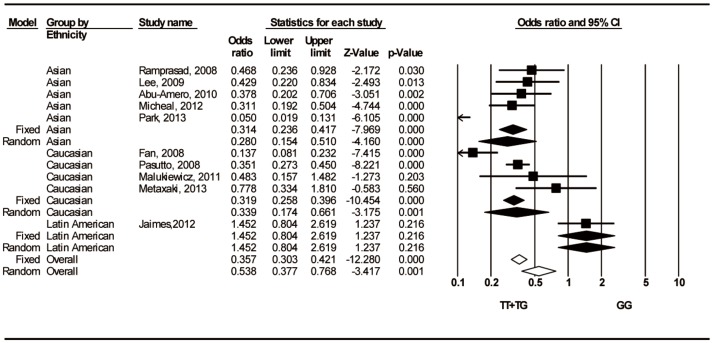
Forest plots for associations between rs1048661 T/G (TT+TG vs. GG) and PEXS/PEXG stratified by ethnicity. The first author of the study and year of publication are shown for each citation. The squares and horizontal lines correspond to the study specific odds ratio (OR) and 95% confidence interval (CI), respectively; the diamonds represent the pooled OR and 95% CI.

Twelve studies evaluated the association between the rs1048661 T/G polymorphism and the risk of developing PEXS/PEXG. For the combined group data, significant associations between rs1048661 and susceptibility to PEXS/PEXG were observed for the additive, dominant, and recessive models, but not for the allele frequency comparison (Table 2). In a subgroup analysis performed by ethnicity, the pooled OR for the Caucasian population indicated a significantly decreased risk for PEXS/PEXG in the dominant (Figure 2), recessive, and TT versus GG additive models (Table 2). For the Asian population, the dominant (Figure 2) and two additive models showed a significant association between rs1048661 and a reduced susceptibility to PEXS/PEXG, while the allele frequency comparison and recessive model did not (Table 2). No significant associations were found in the Latin American population.

**Table 2:** Summary of pooled odds ratios for the association of rs1048661 and PEXS/PEXG in the meta-analysis.

Ethnicity	Genetic model		OR (95% CI)	P	Heterogeneity test		Publication bias
					I2	P (Q-test)	P value
Total	allele frequency comparison	T vs. G	0.942 (0.617–1.436)	0.780	97.2	**0.001**	0.400
	additive model	TG vs. GG	0.545 (0.421–0.705)	**0.001**	71.6	**0.001**	0.185
	additive model	TT vs. GG	0.294 (0.149–0.579)	**0.001**	82.9	**0.001**	0.921
	dominant model	TT+TG vs. GG	0.538 (0.377–0.768)	**0.001**	83.7	**0.001**	0.959
	recessive model	TT vs. TG+GG	0.393 (0.196–0.791)	**0.009**	88.1	**0.001**	0.788
Caucasian	allele frequency comparison	T vs. G	0.439 (0.154–1.251)	0.123	96.5	**0.001**	0.859
	additive model	TG vs. GG	0.678 (0.361–1.273)	0.227	67.5	**0.027**	0.146
	additive model	TT vs. GG	0.067 (0.014–0.327)	**0.001**	82.2	**0.001**	0.829
	dominant model	TT+TG vs. GG	0.339 (0.174–0.661)	**0.001**	80.3	**0.002**	0.910
	recessive model	TT vs. TG+GG	0.079 (0.012–0.513)	**0.008**	88.0	**0.001**	0.775
Asian	allele frequency comparison	T vs. G	1.056 (0.262–4.261)	0.939	97.8	**0.001**	0.748
	additive model	TG vs. GG	0.386 (0.283–0.528)	**0.001**	0	0.998	0.439
	additive model	TT vs. GG	0.447 (0.250–0.800)	**0.007**	47.2	**0.099**	0.304
	dominant model	TT+TG vs. GG	0.280 (0.154-0.510)	**0.001**	75.8	**0.002**	0.286
	recessive model	TT vs. TG+GG	0.596 (0.228–1.559)	0.291	48.9	**0.063**	0.236
Latin American	allele frequency comparison	T vs. G	1.096 (0.673–1.788)	0.712	0	1.000	#
	additive model	TG vs. GG	1.889 (0.989–3.609)	0.054	0	1.000	#
	additive model	TT vs. GG	0.480 (0.141–1.636)	0.241	0	1.000	#
	dominant model	TT+TG vs. GG	1.452 (0.804–2.619)	0.216	0	1.000	#
	recessive model	TT vs. TG+GG	0.399 (0.119–1.342)	0.138	0	1.000	#

CI, confidence interval; I^2^, inconsistency index; OR, odds ratio; PEXG, pseudoexfoliation glaucoma; PEXS, pseudoexfoliation syndrome; SNP, single nucleotide polymorphism; vs., versus. Bold text: significant odds ratio (P<0.05) and significant between-study heterogeneity (P<0.1).

# Publication bias could not be tested because a minimum of 3 studies were required.

#### Association of the rs3825942 A/G polymorphism with PEXS/PEXG

**Figure 3:**
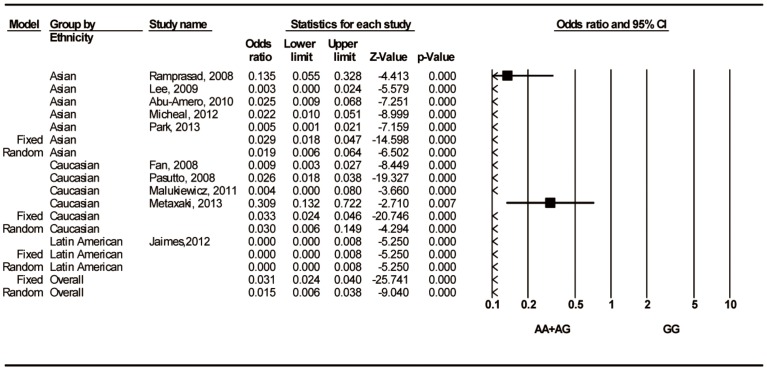
Forest Plots for associations between rs3825942 A/G (AA+AG vs. GG) and PEXS/PEXG stratified by ethnicity. The first author of the study and year of publication are shown for each citation. The squares and horizontal lines correspond to the study specific odds ratio (OR) and 95% confidence interval (CI); the diamonds represent the pooled OR and 95% CI.

The twelve studies contained data regarding the association of the rs3825942 A/G polymorphism with the susceptibility to PEXS/PEXG. In comparison with the control group, the association between rs3825942 and decreased susceptibility to PEXS/PEXG was significant in all genetic models (Table 3). Furthermore, in the subgroup analysis by ethnicity, the genetic models showed a significant association between rs3825942 and a reduced susceptibility to PEXS/PEXG in the Caucasian and Asian populations (Table 3; Figure 3). In the Latin American population, the allele frequency comparison and the dominant model showed significant associations (Table 3) and a reduced risk (Figure 3).

**Table 3:** Summary of pooled odds ratios for the association of rs3825942 and PEXS/PEXG in the meta-analysis.

Ethnicity	Genetic model		OR (95% CI)	P	Heterogeneity test		Publication bias
					I2	P (Q-test)	P value
Total	allele frequency comparison	A vs. G	0.153 (0.104–0.225)	**0.001**	86.6	**0.001**	0.628
	additive model	AG vs. GG	0.153 (0.096–0.244)	**0.001**	42.6	**0.083**	0.296
	additive model	AA vs. GG	0.101 (0.052–0.198)	**0.001**	0	0.933	0.643
	dominant model	AA+AG vs. GG	0.015 (0.006–0.038)	**0.001**	86.4	**0.001**	0.365
	recessive model	AA vs. AG+GG	0.128 (0.066–0.250)	**0.001**	0	0.913	0.653
Caucasian	allele frequency comparison	A vs. G	0.277 (0.075–1.031)	0.056	93.7	**0.001**	0.883
	additive model	AG vs. GG	0.164 (0.057–0.473)	**0.001**	75.5	**0.007**	0.767
	additive model	AA vs. GG	0.085 (0.039–0.189)	**0.001**	0	0.775	0.799
	dominant model	AA+AG vs. GG	0.030 (0.006–0.149)	**0.001**	91.5	**0.001**	0.971
	recessive model	AA vs. AG+GG	0.106 (0.048–0.233)	**0.001**	0	0.777	0.790
Asian	allele frequency comparison	A vs. G	0.148 (0.099-0.223)	**0.001**	0	0.587	0.520
	additive model	AG vs. GG	0.156 (0.092-0.264)	**0.001**	0	0.885	0.068
	additive model	AA vs. GG	0.139 (0.036-0.535)	**0.004**	0	0.778	0.378
	dominant model	AA+AG vs. GG	0.019 (0.006-0.064)	**0.001**	82.1	**0.001**	0.185
	recessive model	AA vs. AG+GG	0.192 (0.050-0.738)	**0.016**	0	0.703	0.420
Latin American	allele frequency comparison	A vs. G	0.048 (0.003–0.826)	**0.036**	0	1.000	#
	additive model	AG vs. GG	0.058 (0.003–1.034)	0.053	0	1.000	#
	additive model	AA vs. GG	0.291 (0.012–7.235)	0.452	0	1.000	#
	dominant model	AA+AG vs. GG	0.001 (0.001–0.008)	**0.001**	0	1.000	#
	recessive model	AA vs. AG+GG	0.314 (0.013–7.797)	0.480	0	1.000	#

CI, confidence interval; I2, inconsistency index; OR, odds ratio; PEXG, pseudoexfoliation glaucoma; PEXS, pseudoexfoliation syndrome; SNP, single nucleotide polymorphism; vs., versus. Bold text: significant odds ratio (P<0.05) and significant between-study heterogeneity (P<0.1).

# Publication bias could not be tested because a minimum of 3 studies were required.

#### Association of the rs2165241 C/T polymorphism and PEXS/PEXG

**Figure 4:**
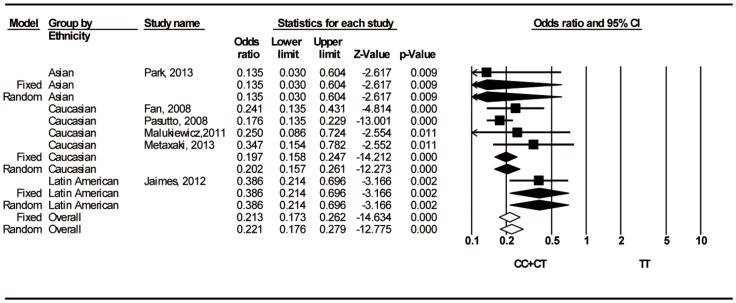
Forest Plots for associations between rs2165241 C/T (CC+CT vs. TT) and PEXS/PEXG stratified by ethnicity. The first author of the study and year of publication are shown for each citation. The squares and horizontal lines correspond to the study specific odds ratio (OR) and 95% confidence interval (CI); the diamonds represent the pooled OR and 95% CI.

Eight studies contained data for the rs216524 C/T polymorphism. The association between rs216524 and susceptibility to PEXS/PEXG was significant in all genetic models (Table 4). Furthermore, the subgroup analysis showed that all ethnicities had significant associations for all of the genetic models (Table 4) and reduced susceptibility to PEXS/PEXG (Figure 4).

**Table 4:** Summary of pooled odds ratios for the association of rs2165241 and PEXS/PEXG in the meta-analysis.

Ethnicity	Genetic model		OR (95% CI)	P	Heterogeneity test		Publication bias
					I2	P (Q-test)	P value
Total	allele frequency comparison	C vs. T	0.661 (0.473–0.924)	**0.015**	95.0	**0.001**	0.212
	additive model	CT vs. TT	0.365 (0.252–0.530)	**0.001**	63.1	**0.029**	0.085
	additive model	CC vs. TT	0.101 (0.073–0.139)	**0.001**	0	0.807	0.603
	dominant model	CC+CT vs. TT	0.213 (0.173–0.262)	**0.001**	37.2	0.159	0.294
	recessive model	CC vs. CT+TT	0.204 (0.152–0.273)	**0.001**	0	0.675	0.338
Caucasian	allele frequency comparison	C vs. T	0.437 (0.199–0.958)	**0.039**	94.6	**0.001**	0.581
	additive model	CT vs. TT	0.246 (0.194–0.312)	**0.001**	45.1	0.106	0.129
	additive model	CC vs. TT	0.094 (0.066–0.133)	**0.001**	0	0.939	0.884
	dominant model	CC+CT vs. TT	0.197 (0.158–0.247)	**0.001**	7.6	0.355	0.105
	recessive model	CC vs. CT+TT	0.200 (0.146–0.274)	**0.001**	0	0.527	0.338
Asian	allele frequency comparison	C vs. T*	6.650 (2.915–15.17)	**0.001**	0	1.000	#
	dominant model	CC+CT vs. TT&	0.135 (0.030–0.604)	**0.009**	7.6	0.355	#
Latin American	allele frequency comparison	C vs. T	0.415 (0.275–0.628)	**0.001**	0	1.000	#
	additive model	CT vs. TT	0.517 (0.276–0.969)	**0.040**	0	1.000	#
	additive model	CC vs. TT	0.163 (0.065–0.409)	**0.001**	0	1.000	#
	dominant model	CC+CT vs. TT	0.386 (0.214–0.696)	**0.002**	0	1.000	#
	recessive model	CC vs. CT+TT	0.232 (0.162–0.307)	**0.001**	0	1.000	#

CI, confidence interval; I2, inconsistency index; OR, odds ratio; PEXG, pseudoexfoliation glaucoma; PEXS, pseudoexfoliation syndrome; SNP, single nucleotide polymorphism; vs., versus. Bold text: significant odds ratio (P<0.05) and significant between-study heterogeneity (P<0.1).

# Publication bias could not be tested because a minimum of 3 studies were required.

* Only allele frequency data were extracted from Ozaki et al. (2008).

& The data for minor homozygosity+heterozygosity versus major homozygosity were extracted from the study of Park et al.

### Between-study heterogeneity analysis

The Q-test suggested significant between-study heterogeneity for several of the pooled models for each of the SNPs (Tables 2, 3, and 4). To examine the possible sources of heterogeneity, the studies were stratified by ethnicity, but the inconsistency index did not substantially decrease. We therefore performed a meta-regression analysis to assess the influence of the study characteristics on the effect estimates.

**Figure 5:**
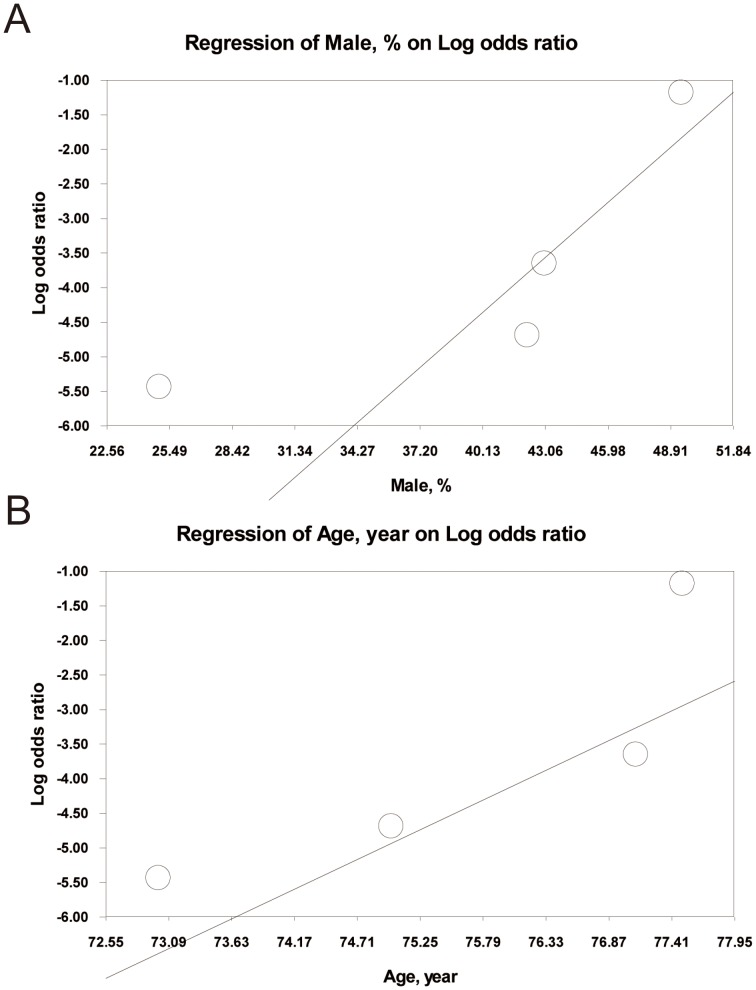
Association between study population characteristics and effect of rs3825942 on PEXS/PEXG susceptibility in Caucasian patients.

The male proportion and mean age of the subjects did not significantly affect the influences of rs1048661 and rs2165241 on PEXS/PEXG susceptibility. In contrast, for the effects of rs3825942 on PEXS/PEXG risk in Caucasians, a significant influence was detected for both the male proportion (Figure 5A; slope: 0.272; 95% CI: 0.167–0.376; P = 0.0001) and mean age (Figure 5B; slope: 0.796; 95% CI: 0.375–1.217; P = 0.0002) of the subjects.

### Sensitivity analyses

Although the genotype distribution in four of the studies did not follow Hardy-Weinberg equilibrium [16], [27], [28], [29], the corresponding pooled ORs were not materially altered with or without including these studies (data not shown). In addition, the sensitivity analyses indicated that no single study had undue influence on the pooled OR results.

### Publication bias

A Begg funnel plot analysis and Egger tests were used to access the publication bias of the literature. The shapes of the funnel plots of dominant models for the SNPs did not reveal evidence of obvious asymmetry (Figure S1).The Egger test results suggested that no publication bias was found in any of the comparison models (Tables 2, 3, and 4).

## Discussion

PEXS/PEXG is a disorder characterized by the accumulation of abnormal fibrillar deposits in the anterior segment of the eye. LOXL1, which serves both as a cross-linking enzyme and an element of the scaffold to ensure spatially defined deposition of elastin and collagen substrates [7], was recently identified by genetic linkage studies as associated with a susceptibility to PEXS/PEXG [14], [15], [31]. A large number of high-frequency SNPs have been identified for this gene [11], [26]. After screening these SNPs with the selection criteria, the rs1048661 T/G, rs3825942 A/G, and rs2165241 C/T polymorphisms were chosen to examine for their association with PEXS/PEXG susceptibility in this meta-analysis.

Our results indicated that rs1048661 TT carriers had 92.1% and 40.4% less risk of developing PEXS/PEXG than did the controls in Caucasian and Asian populations, respectively, but had no influence on the susceptibility in the Latin American population. Carriers of rs3825942 AA or rs2165241 CC also had significantly less risk of developing PEXS/PEXG than did the non-carriers. Despite the ethnic heterogeneity in the LOXL1 genotypes and the consequent variable susceptibility to PEXS/PEXG, the rs1048661, rs3825942 and rs2165241 SNPs may provide a powerful diagnostic tool to identify subjects who are more likely to develop PEXS/PEXG.

The analyses for heterozygosity of the variants are probably due to chance; the studies with small sample sizes for the minor homozygous alleles in PEXS/PEXG subjects would have insufficient statistical power to detect slight effects, or they may have generated a fluctuated risk estimate. Given this situation, our evaluation of the effects associated with heterozygosity for the variants in our analysis should be interpreted with caution.

In meta-analysis studies, heterogeneity could potentially restrict the interpretation of the pooled estimates, and ethnicity could play a role in determining the heterogeneity among studies. The different allele frequencies among the different ethnicities were a strong cause of the heterogeneity, leading us to do a subgroup analysis stratified by ethnicity; however, this resulted in no substantial decrease in the heterogeneity. Although applying a random effect model allowed us to estimate the effects of the different studies, we also performed a meta-regression to assess the influence of the population characteristics on the effect estimates. The results showed that the male proportion and mean age of the PEXS/PEXG patients positively correlated with the effect of SNP rs3825942 on the PEXS/PEXG susceptibility in the Caucasian population [32]. Although our study cannot explain how LOXL1 interacts with gender and mean age, the correlations between them in the Caucasian population could be the primary cause of the heterogeneity. Considering the LOXL1 SNP genotypes together with these clinical predictors (gender and mean age) may allow for greater accuracy in predicting the probability of developing PEXS/PEXG.

To our knowledge, this study is the first meta-analysis to assess the association of the LOXL1 polymorphisms with PEXS/PEXG. This statistical method increased the power to detect and quantify an effect, and it provided a control for population differences that could lead to spurious associations if there are differences in gene frequency among the groups. Furthermore, this method allowed us to confirm the reliability and stability of the meta-analysis by performing publication bias and sensitivity analyses.

Some limitations of this study should be taken into consideration. First, the study populations were primarily Caucasian and Asian. The subgroup meta-analysis for ethnicity had little or no information for other ethnic groups. Thus, strengthening the statistical power will require more data from other ethnic groups. Second, although we were able to discern a significant association between the LOXL1 polymorphisms and susceptibility to PEXS/PEXG in the Caucasian and Asian populations, the sample size after pooling the existing studies was still relatively small. Third, the lack of available data prevented an adjustment for subgroup factors such as age, gender, and other variables that can interact with genetic factors to influence the marginal association estimates between the SNPs and PEXS/PEXG susceptibility. Should such data become available, a more precise analysis allowing for the adjustment of other covariates such as age, family history, environmental factors, and lifestyle would be feasible.

In summary, despite its limitations, this meta-analysis identified an association between the LOXL1 rs1048661, rs3825942, and rs2165241 polymorphisms and the risk of developing PEXS/PEXG. Future studies with larger sample sizes and additional ethnic groups are required to further clarify the association of the polymorphisms with the incidence of PEXS/PEXG, and these studies should consider gene-gene and gene-environment interactions in the analysis. Such studies should lead to a more comprehensive understanding of the influence of the LOXL1 gene polymorphisms on susceptibility to PEXS/PEXG.

## Supporting Information

Figure S1 **Funnel plot analysis for publication bias.** (A) rs3825942 TT+TG vs. GG; (B) rs3825942 AA+AG vs. GG; (C) rs2165241 CC+CT vs. TT.
(DOC)

Checklist S1 **PRISMA checklist.**
(DOC)
