# Correction: Association between Polymorphisms in Lysyl Oxidase-Like 1 and Susceptibility to Pseudoexfoliation Syndrome and Pseudoexfoliation Glaucoma

**DOI:** 10.1371/journal.pone.0099916

**Published:** 2014-06-04

**Authors:** The PLOS ONE Staff

The third author's name is spelled incorrectly. The correct name is: Hua-Feng Ma. The correct citation is: Tang J-Z, Wang X-Q, Ma H-F, Wang B, Wang P-F, et al. (2014) Association between Polymorphisms in Lysyl Oxidase-Like 1 and Susceptibility to Pseudoexfoliation Syndrome and Pseudoexfoliation Glaucoma. PLoS ONE 9(3): e90331. doi:10.1371/journal.pone.0090331.
