# Supplementary figures and images for: Association between Polymorphisms in Lysyl Oxidase-Like 1 and Susceptibility to Pseudoexfoliation Syndrome and Pseudoexfoliation Glaucoma
Source: PLoS One. 2014 Mar 6;9(3):e90331. doi: 10.1371/journal.pone.0090331 (PMC3946061; doi:10.1371/journal.pone.0090331)

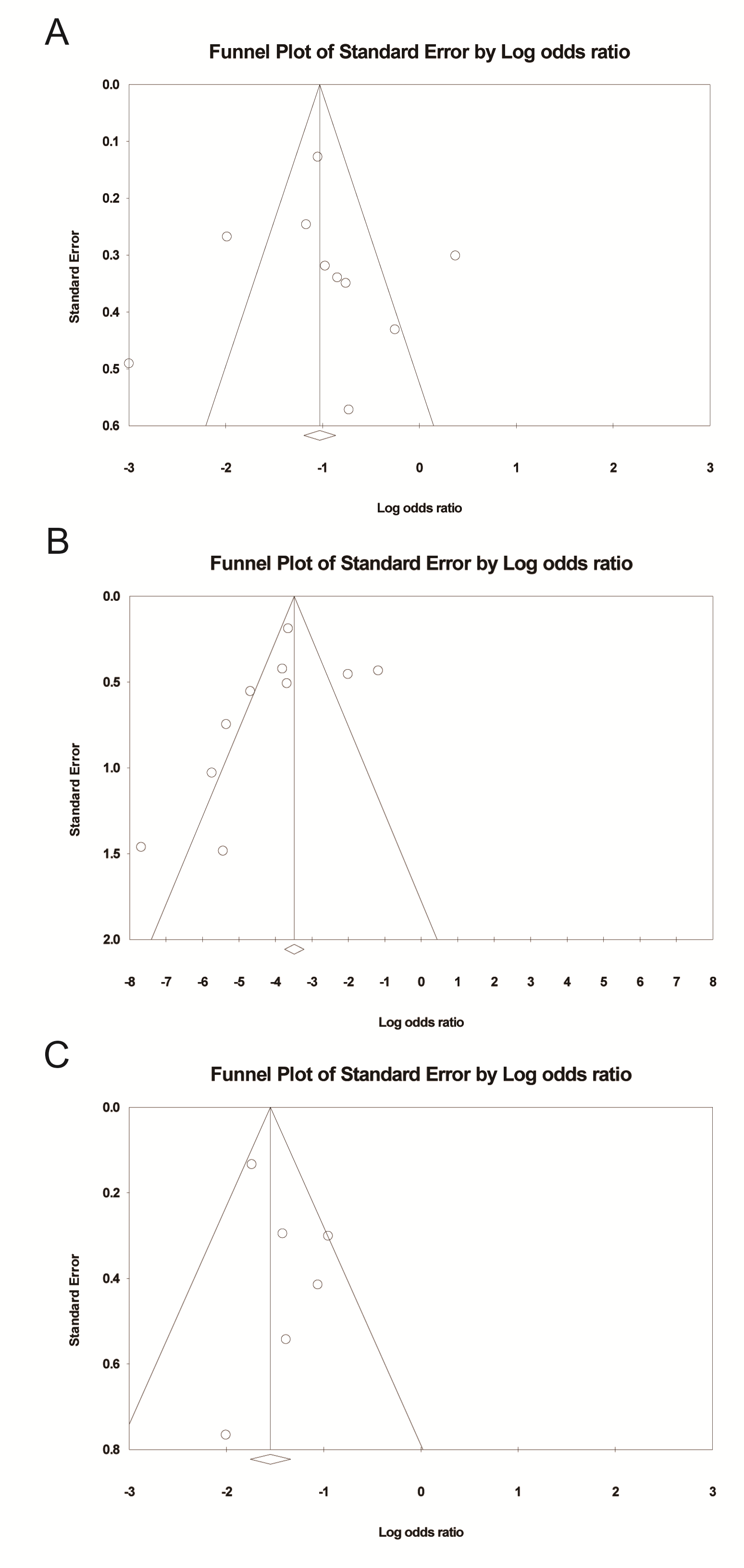

Supplement: Figure S1 — Funnel plot analysis for publication bias. (A) rs3825942 TT+TG vs. GG; (B) rs3825942 AA+AG vs. GG; (C) rs2165241 CC+CT vs. TT. (DOC) [file pone.0090331.s001.doc]
